# Evaluating the impact of social franchising on family planning use in Kenya

**DOI:** 10.1186/s41043-016-0056-y

**Published:** 2016-06-18

**Authors:** Nirali M. Chakraborty, Mwende Mbondo, Joyce Wanderi

**Affiliations:** 1 Metrics for Management, Baltimore, MD USA; 2 Nairobi, Kenya; 3 Population Services Kenya, Nairobi, Kenya

## Abstract

**Background:**

In Kenya, as in many low-income countries, the private sector is an important component of health service delivery and of providing access to preventive and curative health services. The Tunza Social Franchise Network, operated by Population Services Kenya, is Kenya’s largest network of private providers, comprising 329 clinics. Franchised clinics are only one source of family planning (FP), and this study seeks to understand whether access to a franchise increases the overall use or provides another alternative for women who would have found FP services in the public sector.

**Methods:**

A quasi-experimental study compared 50 catchment areas where there is a Tunza franchise and no other franchised provider with 50 purposively matched control areas within 20 km of each selected Tunza area, with a health facility, but no franchised facility. Data from 5609 women of reproductive age were collected on demographic and socioeconomic status, FP use, and care-seeking behavior. Multivariate logistic regression, with intervention and control respondents matched using coarsened exact matching, was conducted.

**Results:**

Overall modern contraceptive use in this population was 53 %, with 24.8 % of women using a long-acting or permanent method (LAPM). There was no significant difference in odds of current or new FP use by group, adjusted for age. However, respondents in Tunza catchment areas are significantly more likely to be LAPM users (adj. OR = 1.49, *p* = 0.015). Further, women aged 18–24 and 41–49 in Tunza catchment areas have a significantly higher marginal probability of LAPM use than those in control areas.

**Conclusions:**

This study indicates that access to a franchise is correlated with access to and increased use of LAPMs, which are more effective, and cost-effective, methods of FP. While franchised facilities may provide additional points of access for FP and other services, the presence of the franchise does not, in and of itself, increase the use of FP in Kenya.

## Background

Health services are, and have been, delivered through a number of different channels including the public sector, private for profit sector, faith-based organizations, community health workers, and mobile services. As shown by a wealth of research over the past 20 years, private sector provision of health care is a significant source of care for both rich and poor families in low- and middle-income countries [1–6]. Effective engagement of the private sector has been promoted as a way to increase access to and coverage of essential health services [7–10].

One intervention designed to improve provision of services in the private sector is social franchising [11–13]. Worldwide, there are at least 64 franchised networks in 35 countries, providing branded, quality-assured services from 39,000 providers [14]. The evidence base for franchising has been building over time, with many studies focusing on outcomes based upon 5 goals agreed upon by a global community of practice for social franchising: quality, health impact, equity, cost-effectiveness, and market expansion [14, 15]. A 2009 Cochrane review found no studies of sufficient rigor to meet their inclusion criteria, while a 2011 review identified 9 studies of varying designs, assessing aspects of access to services, quality of care, impact of franchising on behavior, and equity analyses [16, 17]. This review found mixed outcomes with regard to changes in unmet need for family planning (FP). Recently, Beyeler and colleagues reviewed 23 studies and find some positive evidence that franchising increases knowledge and the use of health services, mixed evidence on FP quality, and that the franchising intervention served to increase client volume at clinics [15]. As with previous reviews, authors found that extant research is still limited and of low quality with regard to the use of rigorous research methodologies.

Social franchising has been used to promote FP in the private sector, in order to increase access to and quality of services. In particular, franchisors have promoted long-acting reversible contraception (LARC) and permanent methods (PMs) in the context of informed choice, as these methods are highly effective and cost-effective yet underutilized [13, 18–20]. Their availability expands the range of methods available to women and reduces discontinuation rates [21]. Yet, the use of LARCs has been historically low.

Outside of the franchising context, several studies have shown that interventions also used by franchisors, such as training, supportive supervision, and job aids, do increase the quality of FP service provision [22, 23]. Similarly, expanding access to FP, through task-sharing, community distribution, or supply improvements, has been shown to increase the use of contraception [24–26]. However, social franchising, as an intervention to increase the use of contraception, has demonstrated mixed results. For example, a study in Nepal showed a marginally significant effect of franchising on FP use, while one in Vietnam did not show any association between attending a franchised clinic and FP use [27, 28]. In contrast, multi-country data from Marie Stopes International show that a large proportion of FP clients have never used FP before, which could be associated with increased access [29].

In light of the previous research, and continued donor and implementer interest in franchising as a way of improving private sector service delivery, and access to needed health services, this study seeks to assess if franchising increases the overall use of family planning services in Kenya, by comparing new users of FP in areas with and without a franchised facility. The study further explores differences in the types of family planning methods used, by women who have access to franchised facilities as compared to those who do not.

### Context

Contraceptive prevalence in Kenya has been increasing over the last 10 years, from 31.5 % in 2003 to 53.2 % in 2014 (modern method used among married women), with more rapid progress over the last 5 years than in the past [30]. Unmet need has declined, from 27.5 to 17.5 % among married women, but inequities by education and wealth are substantial. The use of LARCs has increased among married women, from 3.5 % in 2008 to 13.3 % in 2014, while prevalence of female sterilization has reduced from 4.8 to 3.2 %. LARCs, like all family planning services, are free in the public sector in Kenya. Nevertheless, the role of various service delivery channels in the increase in LARC utilization is unclear.

Kenya has one of the more developed private health care sectors in sub-Saharan Africa, with social franchising networks in existence since 2000 [31]. Several organizations have been able to organize the private sector into networks of facilities that focus on specific health areas. These networks are known as social franchises and include the following: Amua managed by Marie Stopes International, Gold Star Network managed by FHI360, Tunza managed by Population Services Kenya, Huduma Poa Network managed by Kisumu Medical and Education Trust (KMET), and Family Health Option Kenya and CFW managed by Sustainable Healthcare Foundation. Each network is a fractional franchise, focusing on some of the services provided by the facilities. Family planning services are the key for Amua, Tunza, CFW, FHOK, and Huduma Poa while the Gold Star Network focuses on human immunodeficiency virus (HIV) care and treatment services.

Tunza was established in 2009 to increase access to family planning services. It currently comprises 329 clinics in all 8 provinces of Kenya and is positioned to clients as providing friendly, quick, affordable health services by qualified health providers. Seventy-eight percent of Tunza providers are nurses, while the remainders are clinical officers (21 %) and medical doctors (1 %). Clinics are branded with a distinctive purple logo, and 41 % are located in rural areas. Franchise providers receive contraceptive technology updates, training in the Integrated Management of Childhood Illnesses (IMCI), and HIV counseling and testing. Additional health areas that franchise facility providers are trained on, depending on suitability, include safe motherhood, cervical cancer screening and treatment, prevention of mother to child transmission of HIV (PMTCT), and provision of anti-retroviral therapy (ART) services. They are also periodically visited by Population Services International (PSI) staff for quality assurance and clinical updates. Those providing cervical cancer screening and treatment are also provided with commodities and cryotherapy machines to support the service while those providing HIV care and treatment services are linked to the national laboratory grid for their clients to access critical CD4 and viral load testing at subsidized rates. Further, the ART drugs are provided through the national supply chain KEMSA, thereby ensuring continuity of supply. As a part of the franchise package of interventions, PS Kenya also provides franchisees with supported demand creation, employing 14 demand creation supervisors who canvas the community around the clinic for potential clients of the Tunza clinics. On their part, franchise providers are required to provide monthly reports on service provision to PS Kenya as well as the government health management information system for accountability.

## Methods

### Intervention

This study assesses if geographic access to a Tunza-franchised clinic increases the use of family planning. Using a quasi-experimental post-test design with post hoc matching, women residing near Tunza clinics were compared to those residing near public sector clinics. Data was collected via a cross-sectional, population-based survey, sampling among women of reproductive age (18–49) who lived within the control and intervention clusters.

### Study design

Control and intervention clusters were defined based upon a facility (Tunza franchised or non-franchised) as the centroid of a catchment area. A facility catchment area was defined as 1 km for facilities classified as being in urban areas and 3 km for facilities classified as being in rural areas. All Tunza facilities were mapped, and any facility which had another franchise facility within its catchment area, or overlapping with its catchment area, was dropped from the sampling frame. Franchised facilities mapped for the sample included Tunza, MSI Blue Star, and FHI Gold Star facilities. From the 102 “eligible” Tunza facilities, 50 were randomly chosen.

Control catchment areas were chosen subsequently, by listing all public sector facilities within 5–20 km of the Tunza facilities and dropping any facility whose catchment area overlapped with that of a franchise. Eligible public sector facilities included dispensary, health center, medical clinic, and sub-district hospital. From the list of eligible control facilities, one facility was purposively matched to each Tunza facility, considering variables such as facility types, urban or rural location, and distance to the Tunza facility. Priority was given to matching facility types, then to urban or rural location, and finally to selecting the closest facility among those available. Situations where the same facility could serve as a control for multiple Tunza facilities had to be assessed separately, so matching was done by hand.

### Sampling

Sample sizes for the study were calculated based upon the primary hypothesis that households within a franchised cluster would have higher proportion of new users of FP in the last 2 years than those in a control cluster. Data from the 2008 KDHS and a PSI population-based survey from 2011 provided information to estimate the proportion of new users in the last 2 years in the population as 3 % [32]. The study was designed to detect a 2 percentage point difference, with 80 % power and a 95 % confidence interval, accounting for increased homogeneity within clusters (DEFF = 1.7). This yielded an effective sample of 5120, which was rounded up to 5200 sexually active women of reproductive age. In order to ensure an adequate sample size of sexually active women when sampling households, the sample was increased by 10 %; a sample size of 5720 households was sought.

From each of the 100 catchment areas, approximately 60 households were approached for interview. Households were selected using a systematic approach after a random start, with interview teams starting from the health center, walking in opposite directions, and interviewing every third household until reaching the catchment area boundary. Teams then turned left along the boundary, walked for a few minutes, and tried to return back to the health center interviewing every third household.

### Outcomes

The primary outcome of this study was a difference in the new use of family planning. This was assessed by asking current users of modern family planning methods if they had ever used FP prior to November 2011 (2 years prior to the time the survey was implemented). Those who answered no were considered to be new users of FP. Secondary outcomes of this study included differences in the current use of modern family planning and differences in the use of LARCs and PMs.

### Analysis

All three outcomes were assessed using logistic regression, comparing women living in a Tunza catchment area with those living in a control area. Previous studies in Kenya have identified several individual and household characteristics associated with the use of and need for modern family planning [33, 34]. These include age, parity, marital status, education, religion, location, and wealth. We assessed the differences in distribution of these characteristics between the two groups and considered them for inclusion in the study as explanatory variables. By matching instead of including these characteristics in the multivariate models, we are able to generalize our findings to women of reproductive age, living near our facility catchment areas, rather than to those women in a specific wealth, marital, or educational stratum. Other variables which were assessed included number of FP methods known and knowledge of individual methods. These were not considered relevant for inclusion in final multivariate models. Parity was not included in final models due to high correlation with age and no significant difference in the tests of model fit. Each outcome was also assessed to see if age modified the effect of study group on family planning use.

Final models can be described by the following equations, where *P*_*ij*_ represents the probability of a positive outcome for individual *i* in cluster *j* and *T* is a binary variable representing Tunza or control cluster. Age was included as a categorical variable.

$$ \ln \left(\frac{P_{ij}}{1-{P}_{ij}}\right)={\beta}_0+{\beta}_1{T}_{ij}+{\beta}_2\mathrm{Age}{1}_{ij}+\dots +{\beta}_5\mathrm{Age}{4}_{ij}+{\varepsilon}_{ij} $$

$$ \ln \left(\frac{P_{ij}}{1-{P}_{ij}}\right)={\beta}_0+{\beta}_1{T}_{ij}+{\beta}_2\mathrm{Age}{1}_{ij}+\dots +{\beta}_5\mathrm{Age}{4}_{ij}+\dots +{\beta}_6\mathrm{Age}{1}_{ij}*{T}_{ij}+\dots +{\beta}_9\mathrm{Age}{4}_{ij}*{T}_{ij}+{\varepsilon}_{ij} $$

Weighted descriptive analyses and logistic regressions were used in an intention to treat approach, with standard errors adjusted for survey design. Model fit was assessed using a goodness-of-fit test specific to survey sample data, and Wald tests, to select the most parsimonious model [35]. STATA 12 statistical software was used to perform analyses.

In order to obtain information on socioeconomic status, we benchmarked the study population against the 2008 Kenyan Demographic and Health Survey (DHS) wealth index [32]. The wealth index was created as a proxy for long-term standard of living and incorporates a list of items including items such as household ownership of consumer goods, dwelling characteristics, water source, toilet facilities, and other characteristics.

Given the robust presence of franchises throughout Kenya, finding true comparable control areas, or designing a prospective study, was not feasible. Control clusters were purposively selected to be similar to intervention clusters, while not containing any franchise. However, descriptive analyses of the data indicated that the two groups were significantly different on several factors relevant to the use of family planning. This may be a result of selection bias in the placement of private providers or private providers eligible for and participating in a franchised network. In order to reduce observable differences between the control and intervention populations, coarsened exact matching was used [36, 37]. Respondents were matched on wealth quintile, marital status, religion, education, and province in which the facility was located, to make the two groups as similar as possible. The L1 multivariate distance measures overlap between the two distributions, and ranges from 0 to 1, with 1 indicating that the two distributions of data are completely separated and 0 indicating that they overlap exactly [36]. Prior to matching, imbalance between the two groups was 0.26. After matching, imbalance was reduced to 6.5e-16, and 5269 of the 5609 cases were matched. Matching variables noted above were not overly coarsened—each category (wealth quintile, marital status-single, husband living with her, husband living elsewhere, education level-none, primary, secondary, higher, and religion-Catholic, Protestant, Muslim, other) was treated as a bin into which respondents were sorted. Data were weighted based on the number of observations falling into each stratum of the matching procedure. Data were further weighted based upon the population of the location in which the facility was located, in order to be geographically representative. Location is a Kenyan geographic classification, within county, within province.

## Results

A total of 5609 households with an eligible female respondent between 18 and 49 years old were surveyed, and 5606 women were included in the analysis. Table 1 displays the demographic characteristics for both the matched (*n* = 5269) and unmatched (*n* = 5606) samples. In the full sample, there are significant differences between respondents in the control and Tunza areas with regard to average age, wealth, level of education, and religion, leading to including some of these variables in the matching procedure. Only matched results are presented henceforth. The average age of respondents was 28.5 years for women in the Tunza areas and 29.8 years for women in the control areas. The majority of women in the study (80.6 % of cases and 82.9 % of controls) fell into the fourth wealth quintile, indicating they were of higher socioeconomic status. 69.6 % of Tunza area women and 74 % of control area women were Protestant, and both groups had less than a high school education (52.4 % of cases and 54.8 % of controls). The average age of sexual debut was age 18 for both Tunza and control groups. The majority of women were married or living with their partners (70.4 % of cases, 69.5 % of controls). Women in the Tunza areas reported an average of 2.7 live births, while women in the control areas reported an average of 3.1 live births (*p* = 0.039).

**Table 1:** Demographic characteristics of respondents

	Full sample *n* = 5609			Matched sample *n* = 5269		
	Control	Tunza	*p* value	Control	Tunza	*p* value
Sample size	2829	2780	–	2731	2538	–
Age (mean)	30.12	28.37	0.001	29.86	28.52	0.010
Age						
18–24	30.85	35.03	<0.001	31.19	34.38	0.003
25–30	27.77	34.41	–	28.86	34.33	–
31–40	27.84	22.81	–	27.09	23.12	–
41–49	13.53	7.75	–	12.86	8.18	–
Wealth quintile						
Q3	18.45	7.45	<0.001	9.04	7.66	0.655
Q4	80.22	76.09	–	82.93	80.59	–
Q5	1.33	16.46	–	8.03	11.75	–
Education						
None	7.08	3.98	0.017	4.81	3.64	0.741
Primary	56.28	47.20	–	50.04	48.73	–
Secondary	28.60	35.61	–	33.24	35.06	–
Higher	8.03	13.21	–	11.91	12.56	–
Religion						
Catholic	25.38	20.31	0.031	20.04	20.13	0.398
Protestant	69.54	66.21	–	74.00	69.56	–
Muslim	4.06	12.12	–	5.22	9.48	–
Other	1.02	1.36	–	0.74	0.83	–
Marital status						
Living together in union	66.66	67.22	0.385	69.54	70.35	0.736
Living apart in union	11.87	9.67	–	10.17	9.01	–
Single	21.48	23.10	–	20.29	20.64	–
Age of sexual debut (mean)	17.85	18.33	0.102	18.19	18.26	0.806
Number of live births (mean)	3.33	2.71	<0.001	3.10	2.73	0.039

Full sample weighted by population size of location; matched sample weighted by population size and stratum; *p* values from *t* tests (for means) or chi^2^ tests (for proportions) comparing control and Tunza groups; standard errors adjusted for clustering by facility catchment area

Table 2 shows family planning characteristics for both matched and unmatched samples. Women who were pregnant were excluded from multivariate analyses, and there was no significant difference in the proportion of women pregnant in the control and Tunza groups. Within the matched sample, 59.6 % of cases reported using some method of family planning, with 53.4 % reporting the use of a modern method. Similarly, 60.2 % of controls reported using some method of family planning, with 53.5 % reporting the use of a modern method. Women in Tunza areas were significantly more likely to be currently using a long-acting or permanent method (LAPM) of family planning than those in control areas (27.3 vs 20.9 %, *p* = 0.028). They were also significantly more likely to have sought their method from a franchised or other private facility (*p* < 0.001), with 16.3 % of current FP users in Tunza areas using Tunza facility, as compared to 1.3 % of women in the control areas.

**Table 2:** Family planning use and knowledge

	Full sample *n* = 5609			Matched sample *n* = 5269		
	Control	Tunza	*p* value	Control	Tunza	*p* value
Currently pregnant	7.14	8.08	0.336	7.90	7.88	0.809
Currently using FP	58.68	57.69	0.751	60.18	59.59	0.861
Currently using modern FP	53.36	51.55	0.532	53.52	53.35	0.955
Currently using LAPM	21.88	26.56	0.071	20.94	27.26	0.028
New user of FP	25.25	27.29	0.560	27.61	27.76	0.968
Source of current FP						
Public sector	77.83	54.36	<0.001	77.60	53.81	<0.001
Franchise	1.58	16.78	–	1.35	16.26	–
Other private	20.12	27.89	–	20.56	29.02	–
Method knowledge (avg # known)	8.18	7.87	0.477	8.18	7.94	0.566
Female sterilization	63.09	55.52	0.179	60.55	55.95	0.372
Male sterilization	43.80	38.89	0.123	43.18	39.11	0.253
IUD	80.32	79.37	0.758	80.92	79.86	0.727
Injectable	92.29	93.51	0.533	92.49	94.03	0.381
Implant	82.67	83.06	0.905	83.34	84.14	0.796
Pill	88.70	89.00	0.916	88.38	89.25	0.748
Male condom	80.04	79.04	0.822	79.64	79.09	0.905
Female condom	63.83	59.35	0.329	64.99	59.85	0.280
SDM	32.71	30.52	0.554	31.93	31.41	0.895
LAM	39.12	35.47	0.423	38.89	36.57	0.596
Rhythm method	52.34	47.71	0.452	51.75	48.18	0.584
Withdrawal	51.21	47.47	0.446	52.43	48.24	0.395
EC	47.80	48.02	0.963	49.18	47.93	0.806

Full sample weighted by population size of location; matched sample weighted by population size and stratum; *p* values from *t* tests (for means) or chi^2^ tests (for proportions) comparing control and Tunza groups; standard errors adjusted for clustering by facility catchment area

When asked, women were familiar with about 8 different modern family planning methods (7.9 for cases, 8.2 for controls). The largest proportion of women, 92.5 and 94 % of control and intervention group women, respectively, were aware of the injectable, while only 60.6 % of control and 56 % of intervention group women were aware of female sterilization. There were no significant differences in method knowledge, and it was not considered relevant for inclusion in multivariate models.

In Table 3, we present results of the multivariate analyses, described by Eq. 1, in the matched sample. Women residing near Tunza-franchised facilities were more likely to use long-acting and permanent methods (OR 1.48, 95 % CI 1.016, 1.957) than women residing in control areas. No statistically significant relationships were found between proximity to Tunza facilities and being a current (OR 0.95, 95 % CI 0.68, 1.21) or new (OR 0.96, 95 % CI 0.59, 1.32) user of modern family planning methods. Age group was significantly associated with all three outcomes.

**Table 3:** Odds of family planning use: AOR and 95 % CI

	Current FP user	Current LAPM user	New user within past 2 years
In Tunza area	0.949	1.486	0.959
	(0.684–1.214)	(1.016–1.957)**	(0.595–1.324)
Age: 18–24 (reference)			
25–30	1.667	1.5	0.378
	(1.325–2.010)***	(0.864–2.137)*	(0.227–0.529)***
31–40	1.586	1.997	0.237
	(1.219–1.954)***	(1.387–2.608)***	(0.139–0.334)***
41–49	0.705	2.47	0.22
	(0.509–0.901)**	(1.883–3.057)***	(0.122–0.319)***
Observations	5195	3198	3198

95 % confidence intervals in parentheses

*Significant at 10 %; **significant at 5 %; ***significant at 1 %

**Fig. 1:**
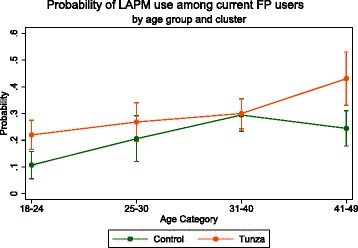
Probability of LAPM use among current FP users, by age group and cluster

We assessed if age modified the effect of study group on family planning use (Eq. 2). Wald tests indicated no significant effect of the interaction on odds of current family planning use (*p* = 0.79) or odds of new family planning use (*p* = 0.69). However, odds of being a current LAPM user differ by age within each group (*p* = 0.036). The probability of LAPM use among current family planning users is shown in Fig. 1. Women in the 18–24 age group in Tunza areas have a significantly greater probability of LAPM use (Pr_Tunza_ = 0.22 vs Pr_control_ = 0.11, *p* < 0.01). There is no significant difference in the use of LAPMs for women 25–30 or 31–40. Women in the 41–49 age group living near a Tunza franchise also have a greater likelihood of LAPM use (Pr_Tunza_ = 0.43 vs Pr_control_ = 0.24, *p* < 0.01).

## Discussion

Despite the popularity of social franchising as an intervention to improve access and quality of private sector health services, evidence of its ability to do so has been limited [15, 16]. This study assessed whether women living near a Tunza social franchise were more likely to be new or current users of family planning, as compared to similar women living near a public sector facility. Living near a Tunza franchise was used as a proxy for access to a franchise. The results from this study indicate that access to a franchise does not lead to greater use of family planning overall but does lead to greater use of LAPMs in comparison with women who live near a public sector facility, but not near a franchise. The results from this study increase the evidence base for how social franchising impacts access to family planning in general and for specific methods and age groups.

Franchising was shown not to have any impact on new or current users of family planning. These findings are consistent with findings from Vietnam and the Philippines, indicating that franchising alone does not increase the use of reproductive or maternal health services [28, 38]. A recent quasi-experimental study in Pakistan introduced an intervention which included training private sector providers and including them in the Suraj social franchise network, providing community mobilization and demand generation for family planning services, and providing vouchers for intra-uterine devices (IUDs) only for clients deemed eligible by a means test. In this study, women living in communities with the new franchised providers were dramatically more likely to use any FP and IUDs specifically [39]. While our study also found increased use of IUDs (and implants) in the intervention areas, no vouchers were available, and demand generation was more limited. Thus the two findings are similar with regard to increased use of IUDs in areas served by franchised clinics, but not directly comparable. While evidence on the ability of franchising to affect general family planning use is mixed, some evidence exists that franchising can impact the use of child health services [40, 41].

Our findings may be explained by a rapid expansion of family planning services throughout Kenya over the past 10 years. Between 2003 and 2014, contraceptive prevalence among married women in Kenya has increased from 39 to 58 % [30]. There has been significant investment in FP by major international donors, including Department for International Development (DFID), United States Agency for International Development (USAID), United Nations Population Fund (UNFPA), and Kreditanstalt fur Wiederaufbau (KFW), as well as access to free commodities to all public and private sector service providers.

Women who did have access to franchised clinics may have been more apt to switch to LAPMs. Our results showed that women living near franchised facilities were significantly more likely to use LAPMs, and further analysis of the data indicates that the difference is driven by the use of implants. Given no difference in overall use, or new users, we conclude that the presence of the Tunza franchise improves access to a wide variety of methods, allowing women’s greater choice in meeting their reproductive intentions.

While the supply side premise of the franchise intervention is to assure commodities, train providers, and support the provision of quality services, the same attention is not always provided in the public sector. Tunza franchised providers have the equipment necessary to provide safe IUD and implant insertions and removals, as well as the necessary commodities. In the public sector, 74 % of facilities (not counting dispensaries or stand alone voluntary counseling and testing (VCT) centers) state that they provide IUDs; however, only 53.5 % had all equipment and commodity available for IUD provision. For implant provision, 62 % of the same public facilities state that they provide the service, and 28 % had all basic items plus method available for implant provision.^1^ Thus, despite high commodity availability, and free or very low-cost service provision to clients, the public sector does not appear adequately equipped to offer women a full range of FP methods.

The increased probability of LAPM use among women aged 18–24 in franchised areas is noteworthy. The use of long-acting methods for younger or nulliparous women is often discouraged by providers, so their ability to seek these methods from Tunza franchises may speak to successful provider training, as well as full service readiness [42, 43]. Both the younger and older women (aged 41–49) may be motivated to visit the franchise sector because of better perceived quality or higher satisfaction or because of the demand generation activities associated with the franchise [44]. The ability to receive highly effective long-term methods from franchises may imply that women are more successful in meeting their reproductive intentions of spacing or limiting births. Franchised providers are not offered any incentives from the franchise network (Tunza) to deliver LAPMs, or any other family planning method, in accordance with US Government regulations enacted to avoid coercion.

Despite a deliberate, rigorous design to isolate the effect of the franchised clinic on the access to and use of family planning services, our study has limitations. By only selecting catchment areas with no other known franchised facility nearby, the sample is more rural than that Tunza network as a whole. There are other private providers, not affiliated with a franchise, or perhaps affiliated with a franchise that we were not aware of, located in the catchment areas of these clusters. We assumed that women would be most likely to go to facilities located close to them; however, this may not always be true, and the study was not able to confirm this assumption. A much higher proportion of women living in the franchised areas, however, received services from the franchised or non-franchised private providers as compared to those in control areas. The results imply that family planning care is shifting away from the public sector in these areas, although the public sector still provides the majority of FP services.

Statistical matching of women in the two groups reduced observable bias; however, unobservable bias may still be impacting the results. We were unable to prospectively assess the impact of the franchise intervention on family planning use and access, which could make this type of an assessment more robust. The quasi-experimental design used here may suffer from threats to internal validity, including the motivations of public vs private sector providers. Nevertheless, it provides useful evidence on how social franchises may influence method choice and use.

## Conclusions

This study demonstrates that in an environment where family planning commodities are widely available, the private sector, and specifically franchised clinics, may be better able to offer a wide range of contraceptive choices to women as compared to the public sector. Women, when offered choice, may be more inclined to seek long-acting methods of contraception, widely considered to be more effective and cost-effective than short-acting methods. Further operations research is required to understand how women’s demand for LARCs and PMs can be successfully met in the public as well as private sectors, as well as how to ensure equitable access to high-quality services, as private sector services are not free.

## Abbreviations

ART, anti-retroviral therapy; DFID, Department for International Development; DHS, Demographic and Health Survey; FP, family planning; HIV, human immunodeficiency virus; IMCI, Integrated Management of Childhood Illness; IUD, intra-uterine device; KFW, Kreditanstalt fur Wiederaufbau; LAPM, long-acting or permanent method; LARC, long-acting reversible contraceptive; PM, permanent method; PMTCT, prevention of mother to child transmission; PSI, Population Services International; UNFPA, United Nations Population Fund; USAID, United States Agency for International Development; VCT, voluntary counseling and testing

## Footnotes

1 Author calculation from data found in Tables A5.10 and A5.12 of the 2010 Kenya Service Provision Assessment.
